# Highly Efficient Near-Infrared Light-Emitting Diodes Based on Chloride Treated CdTe/CdSe Type-II Quantum Dots

**DOI:** 10.3389/fchem.2020.00266

**Published:** 2020-04-17

**Authors:** Huwei Feng, Jiaojiao Song, Bin Song, Qingli Lin, Huaibin Shen, Lin Song Li, Hongzhe Wang, Zuliang Du

**Affiliations:** Key Lab for Special Functional Materials, Ministry of Education, National and Local Joint Engineering Research Center for High-Efficiency Display and Lighting Technology, School of Materials Science and Engineering, Collaborative Innovation Center of Nano Functional Materials and Applications, Henan University, Kaifeng, China

**Keywords:** near-infrared, chloride, CdTe/CdSe, QD, electroluminescence

## Abstract

Quantum dot light-emitting diodes (QLEDs) have been considered as the most promising candidate of light sources for the new generation display and solid-state lighting applications. Especially, the performance of visible QLEDs based on II-VI quantum dots (QDs) has satisfied the requirements of the above applications. However, the optoelectronic properties of the corresponding near-infrared (NIR) QLEDs still lag far behind the visible ones. Here, we demonstrated the highly efficient NIR QLEDs based on chloride treated CdTe/CdSe type-II QDs. The maximum radiant emittance and peak external quantum efficiency (EQE) increased by 24.5 and 26.3%, up to 66 mW/cm^2^ and 7.2% for the corresponding devices based on the chloride treated CdTe/CdSe QDs with the PL peak located at 788 nm, respectively, compared with those of devices before chloride treatment. Remarkably, the EQE of > 5% can be sustained at the current density of 0.3–250 mA/cm^2^ after the chloride treatment. Compared with NIR LEDs based on transition metal complex, the efficiency roll-off has been suppressed to some extent for chloride treated CdTe/CdSe based NIR QLEDs. Based on the optimized conditions, the peak EQE of 7.4, 5.0, and 1.8% can be obtained for other devices based on chloride treated CdTe/CdSe with PL peak of 744, 852, and 910 nm, respectively. This improved performance can be mainly attributed to the chloride surface ligand that not only increases the carrier mobility and reduces the carrier accumulation, but also increases the probability of electron-hole radiative efficiency within QD layers.

## Introduction

Near-infrared (NIR) light-emitting diodes (LEDs) have a rapid development in last decades, because their foreseeable great potential for applications in bio-imaging and clinical diagnosis, night-vision equipments, fiber-optic communications, and computing (Graham et al., 2011; Sun et al., 2012; Dai et al., 2017; Panfil et al., 2018; Song E. et al., 2019). Recently, the peak external quantum efficiency (EQE) of NIR light sources based on transition metal complex (such as osmium, iridium, and platinum), organic LEDs (OLEDs) and perovskite LEDs (PeLEDs) has been up to > 9%, 10% (at 721 nm), and even more than 20% (20.7% at 803 nm, and 21.6% at 800 nm), respectively (Graham et al., 2011; Cao et al., 2018; Kim et al., 2018; Xu et al., 2019). However, for NIR LEDs based on transition metal complex, high costs, relatively scarce resources and the roll-off of efficiency at high brightness remain huge challenges for their large-scale and long-term applications (Wang et al., 2015); organic dyes and semiconductor polymers frequently suffer from the low photoluminescence (PL) quantum yield (QY) in the NIR regime (Gong et al., 2016); perovskite semiconductors often sustain severe trap-mediated non-radiative losses, which have been identified as a major efficiency-limiting factor for LEDs (Xu et al., 2019), moreover, the highly unstable property still hinders their industrialization.

As an alternative material, II-VI semiconductor quantum dots (QDs) demonstrate unique superiorities as NIR emitters, due to high PL QY, easily tunable size-dependent emissions, low-cost solution processability and scalable production of high-quality QDs (Kwak et al., 2012; Shirasaki et al., 2012; Chen et al., 2013; Qin et al., 2013; Zhang et al., 2019). Recent advances in visible LEDs based on II-VI QDs (especially type I structure) have already satisfied the requirements for display and solid-state lighting (Dai et al., 2014; Yang et al., 2015; Zhang et al., 2017; Li et al., 2019; Shen et al., 2019; Song J. et al., 2019). In particular, very recently, the novel strategy that optimizes shell materials to get a better energy level matching with the highest occupied molecular orbital (HOMO) of the hole transport layers facilitates their industrialization (Yang et al., 2015; Li et al., 2019; Shen et al., 2019). However, simple tuning the size of high-quality type-I QDs could not modulate their emission wavelengths to deep-red or near-infrared regions, which restricted their application in NIR fields. Whereas, CdTe/CdSe type-II QDs can be extended into the NIR region easily, along with high PL QY and good stability, due to the higher energies of valence and conduction bands of the core than those of the shell (Kim et al., 2003; Shea-Rohwer et al., 2013), which make them kind of the most promising NIR materials.

Usually, during the synthesis process of QDs, long-chain alkyl ligands are often used to passivate the surface dangling bonds of QDs and facilitate their dispersion in organic solvents with reduced aggregation (Shen et al., 2015). But these long aliphatic ligands act as barriers for charge carrier injection or extraction and lower potential performance of QDs in many optoelectronic devices (Zanella et al., 2013; Page et al., 2014). To solve this issue, on one hand, short-chain thiol ligands [such as 1-octanthiol, tris(mercaptomethyl) and 2-ethylhexane-1-thiol] are used to replace the long-chain ligands by the solution-phase ligand exchange method to improve carrier mobility, which has been demonstrated in the high-performance visible QLEDs (Shen et al., 2015; Li et al., 2016; Song J. et al., 2019). On the other hand, embedding QDs in a high-mobility hybrid perovskite matrix has been demonstrated to be effective by Gong et al. (2016). They achieved a record electroluminescence power conversion efficiency of 4.9% of NIR LEDs (Gong et al., 2016). Recently, the method of the QD surface passivation by inorganic chloride ion has been paid more attention. In 2015, Page et al. reported that Cl passivation on CdTe QDs led to almost total suppression of surface trapping of photogenerated charge carriers and an increase of PL QY from ca. 5% to up to 97.2 ± 2.5% (Page et al., 2014). Recently, Li et al. demonstrated the high brightness QLEDs with low efficiency roll-off by using thionyl chloride (SOCl_2_) to replace the organic ligands capped on the QD surface, in which the chlorination improved the conductivity of QD films and further facilitated the charge carrier injection and mitigated the carrier accumulation (Li et al., 2018). The above results suggest that chloride ion as the ligand not only effectively passivates the dangling bonds on QD surface, but also improves the carrier mobility and suppress the efficiency roll-off. Therefore, the chloride treated QDs as the emissive layers would be expected to enhance the performance of NIR QLEDs.

For this purpose, herein, we demonstrated the highly efficient NIR LEDs based on chloride treated CdTe/CdSe type-II QDs through the all solution-processable procedure. By employing CdTe/CdSe QDs with PL peak at 788 nm as the emitting layer, the maximum radiant emittance and EQE increased from 53 mW/cm^2^ and 5.7% to 66 mW/cm^2^ and 7.2%, respectively, after the chloride treatment. Specially, the EQE of > 5% can be sustained in the range of 0.3–250 mA/cm^2^. Compared to NIR LEDs based on transition metal complex, the efficiency roll-off has been suppressed to a certain extent for chloride treated CdTe/CdSe based NIR QLEDs. Based on the optimized conditions, the maximum EQE of 7.4, 5.0, and 1.8% can also be achieved for other NIR QLEDs based on chloride treated CdTe/CdSe QDs with PL peaks positioned at 744, 852, and 910 nm, respectively. These results may offer new ideas for the further development of NIR QLEDs and facilitate their applications in NIR fields.

## Materials and Methods

### Materials

All reagents were used as received without further experimental purification. Cadmium oxide (CdO, 99.998%), cadmium chloride (CdCl_2_, 99.99%), tellurium powder (Te, 99.8%), tri-n-octylphosphine oxide (TOPO, 90%), oleic acid (OA, 90%), 1-octadecene (ODE, 90%), tri-n-octylphosphine (TOP, 90%), selenium (Se, 99.99%, powder), zinc acetate (99.99%), dimethyl sulfoxide (DMSO, 99.7%), tetradecylphosphoric acid (TDPA, 97%), tributylphosphine (TBP, 90%), tetramethylammoniumhydroxide (TMAH, 97%), and chlorobenzene (99%) were purchased from Aldrich. *n*-Octane (>99%) and isopropanol (99.8%) were purchased from Acros Organics. Hexane (analytical grade), acetone (analytical grade), and methanol (analytical grade) were obtained from Beijing Chemical Reagent Co., Ltd. China.

### Synthesis of Near-Infrared CdTe/CdSe QDs

CdTe/CdSe core/shell QDs were prepared according to the modification of previously reported procedures of our group (Shen et al., 2010, 2013). Details of synthesis are provided in the Supplementary Material.

### Ligand Exchange

0.30 g (1.64 mmol) CdCl_2_ and 0.033 g (0.12 mmol) TDPA were dissolved in 5.0 mL oleylamine and degassed at 120°C for 30 min, then, cooled down to 60°C under N_2_ flow for further reaction.

Five milliliters toluene solution of CdTe/CdSe QDs (15 mg/mL) was degassed at 60°C for 30 min followed by quickly injection into CdCl_2_ stock solution and reacting for 15 min. The as-prepared chloride CdTe/CdSe QDs were purified and dissolved in octane with a concentration of 10 mg/mL.

### Device Fabrication

ZnO nanoparticles were synthesized according to a previous report (Qian et al., 2010). Typically, a stoichiometric amount of TMAH in ethanol (0.5 M) were dropwise added into 0.1 M zinc acetate in DMSO, and stirred for 1 h under ambient atmosphere, and the reaction product of ZnO was then washed and redispersed in ethanol to form a clear solution with a concentration of 30 mg/mL.

QLEDs were fabricated based on the glass substrates with prepatterned ITO, with a sheet resistance of ~15 Ω sq^−1^. The substrates were ultrasonically cleaned with different solvents of deionized water, acetone and isopropanol, each for 15 min. Then the substrates were dried under nitrogen flow and followed by 15 min of UV-Ozone treatment to obtain clean and active surfaces. PEDOT:PSS [Poly(3,4-ethylenedioxythiophene)/poly(styrenesulfonate), AI 4083] was filtrated and spin-coated onto the glass/ITO substrates with spin speed of 5,000 rpm and baked at 150°C for 15 min in air to form a smooth hole injection layer (HIL). These substrates were then immediately transferred into a glove box filled with N_2_ for the further preparation. TFB (poly[9,9-dioctylfluorene-co-N-[4-(3-methylpropyl)]-diphenylamine]) with a concentration of 8 mg/mL (in chlorobenzene) was spin-casted at 3,000 rpm for 30 s, and then baked at 150°C for 30 min to form the hole transport layer (HTL). QDs (10 mg/mL, in octane) and ZnO (30 mg/mL, in ethanol) were sequentially spin-coated onto the layer of TFB at the same spin-speed of 3,000 rpm, followed by a 30 min annealing process at 60°C for each layer. QDs and ZnO were used as the emissive layer and the electron transport layer (ETL), respectively. Finally, these samples were moved into a high-vacuum deposition chamber that located in the glove box to evaporate the 100 nm thick Al cathode. An active device area of 4 mm^2^ was eventually formed with the assistance of an *in situ* shadow mask under a back ground pressure of ~3 × 10^−7^ torr.

### Measurements and Characterization

Room temperature UV-vis absorption were tested by using an UV-vis spectrometer of PerkinElmer Lambda 950. The PL spectra, absolute QY data, and the time-resolved fluorescence spectra of the QDs were recorded by a JY HORIBA FluoroLog-3 fluorescence spectrometer accompanied with an integrating sphere. The optical density (OD) values of the QD samples were set in the range of 0.02–0.05 for UV-vis and PL characterization. Dropcast QD films were deposited on glass substrates for the phase and the crystallographic structure analysis, which was characterized by an X-ray diffractometer (XRD) of Bruker D8-ADVANCE. Transmission electron microscopy (TEM) studies were performed using a JEOL JEM-2100 with an accelerating voltage of 200 kV. X-Ray photoelectron spectroscopy (XPS) was obtained with a monochromatic Al Kα source, 15 kV/8 mA using a Kratos Axis-Ultra spectrometer.

The current density-voltage-radiance characteristics for the QLEDs were measured using a Keithley 2400 source meter and a picoammeter (Keithley 6485) with a calibrated Newport silicon diode under ambient conditions. The electroluminescence spectra were powered by a Keithley 2400 source meter and were recorded by an Ocean Optics USB2000 spectrometer. The EQE is calculated according to the published reference (Forrest et al., 2003), as presented Equation (2).

## Results and Discussion

High-quality CdTe/CdSe type-II QDs were synthesized according to previously reported method. QDs with different emission wavelength could be obtained by coating different thickness of CdSe shells onto the CdTe cores. Supplementary Figures 1A–D shows the absorbance and PL spectra of as-prepared type-II CdTe/CdSe QDs with PL peaks at 744, 788, 852, and 910 nm, accompanied by the PL QYs of ~73, ~69, ~66, and 65%, respectively. According to the TEM images (Supplementary Figures 1E–H), the average sizes are estimated to be ~4.6, ~6.7, ~7.6, and ~9.7 nm, respectively. HRTEM images (insets of Supplementary Figures 1E–H) show the clear lattice fringes throughout the whole QDs, indicating the high crystallinity nature of such type-II CdTe/CdSe QDs. In order to further explore the evolution of crystal structures of CdTe/CdSe core/shell QDs, their phase and crystallographic properties were investigated by XRD (Supplementary Figure 2). As can be seen from the XRD patterns, the diffraction peak positions of the QDs with PL peak located at 744 nm are in good agreement with the structure of bulk cubic CdTe. With the continuous growth of CdSe shell, a small diffraction peak shift to the reference peaks of cubic CdSe could be observed, which was accompanied by a redshift of PL emission.

**Figure 1:**
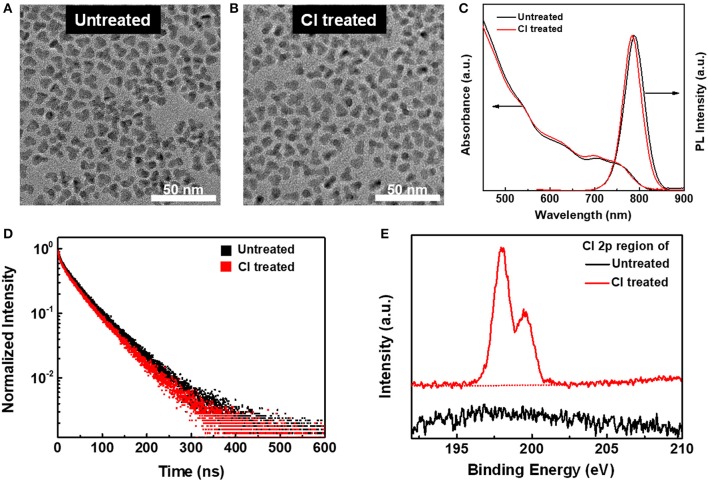
**(A,B)** TEM images of CdTe/CdSe QDs with PL peak at 788 nm **(A)** before and **(B)** after chloride treatment. **(C)** UV-visible absorption and PL spectra of CdTe/CdSe (λ_ex_ = 430 nm) before and after chloride treatment. **(D)** PL decay dynamics of CdTe/CdSe QDs before and after chloride treatment. **(E)** Cl 2p XPS of CdTe/CdSe QDs before chloride treatment and after chloride treatment.

Taking the CdTe/CdSe QDs with PL peak at 788 nm as an example, we adopted chloride ions to modify the QD surface, aimed to improve the electroluminescence performance of NIR QLEDs. In Figures 1A,B, TEM images show no obvious change in the size and morphology before and after chloride treatment. Figure 1C shows the UV-vis absorption and PL spectra of untreated and chloride treated CdTe/CdSe QDs. After chloride treatment, the absorbance spectra exhibit no obvious change, indicating that there is little net effect on the band gap (Li et al., 2016). While, the PL peak has a blue shift of ~3 nm, and full width at half maximum (FWHM) of PL narrowed to 46 nm. Moreover, the PL QY of chloride treated CdTe/CdSe still sustained 60%, which indicates that chloride modification on the QD surface have no obviously negative influence on the PL properties. Correspondingly, the PL QYs of ~65, ~59, and ~57% is for the chloride treated QDs with PL peak of 744, 852, and 910 nm, respectively. This result is also supported by the PL decay dynamics of CdTe/CdSe QDs before and after chloride treatment in Figure 1D. The PL decay of CdTe/CdSe QDs could be well fitted into a tri-exponential decay function, including the fast PL decay (τ_1_) from the recombination of band-edge exciton states, moderate PL decay (τ_2_) resulted from the shallow trap-assisted exciton recombination, and long PL decay (τ_3_) related to deep trap-assisted exciton recombination. The average PL lifetime of CdTe/CdSe QDs just reduced from 58.4 to 54.7 ns. As shown in Supplementary Table 1, for chloride treated CdTe/CdSe QDs, the fraction of long PL lifetime (τ_2_ and τ_3_) caused by the surface traps showed no dramatical decrease compared to untreated CdTe/CdSe QDs. This result suggests that few of new surface traps were introduced into QDs during the process of chloride treatment. XPS were used to confirm the existence of chloride ions on the surface of the treated CdTe/CdSe QDs. As shown in Figure 1E, upon chloride treatment, a Cl 2p doublet peak appears in the spectrum, which is absent for the untreated samples.

**Figure 2:**
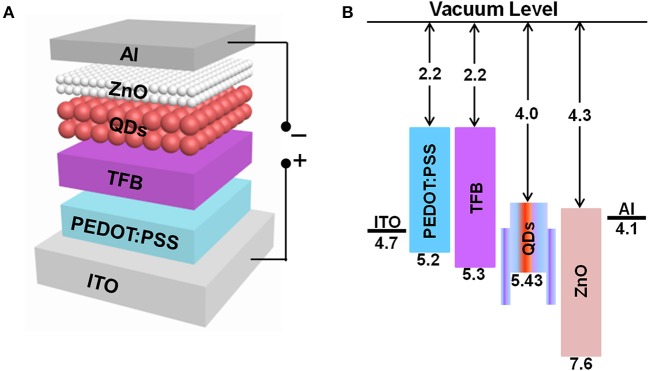
**(A)** The schematic representation of all-solution-processed NIR QLEDs. **(B)** Energy band diagram of QLEDs. The energy values of type-II QDs are cited from Lo et al. (2011).

To evaluate the performance of QLEDs based on chloride treated and untreated CdTe/CdSe QD emitters, multilayered solution-processable device architecture shown in Figure 2A were employed, details for device fabrication were described in the previous section. Based the energy band diagram shown in Figure 2B, TFB is chosen as the HTL due to its relatively low HOMO level of 5.4 eV and high hole mobility of 1.0 × 10^−2^ cm^2^ V^−1^ s^−1^, which can facilitate the hole injection and transport (Choulis et al., 2005). With an electron affinity of ~4.3 eV and an ionization potential of ~7.6 eV (Qian et al., 2011), ZnO was chosen as ETL, consequently facilitates both the electron injection from the cathode and impedes the hole transport to the cathode. Such a structure was designed to increase the probability of hole-electron recombination within the QD layer.

**Figure 3:**
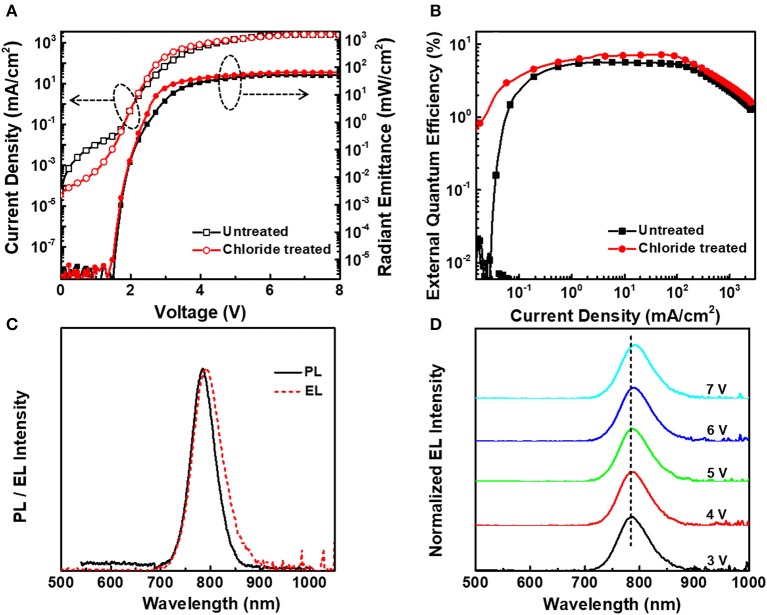
**(A)** Characteristics of current density-voltage-radiant emittance of CdTe/CdSe with PL peak at 788 nm based NIR QLEDs before and after chloride treatment. **(B)** Dependence of EQE on the current density of the corresponding devices. **(C)** PL spectrum of CdTe/CdSe QD solution and the corresponding EL spectrum of the device. **(D)** The evolution of EL spectra of NIR QLEDs with increasing driving voltage.

Figure 3 characterizes the optical-electronic performance of NIR QLEDs based on chloride treated and untreated CdTe/CdSe type-II QDs. In Figure 3A, both of devices exhibit a low turn-on voltage (at which the optical signals can be completely responded by the picoammeter (Keithley 6485) with a calibrated Newport silicon diode) of ~1.5 V. After chloride treatment, the device exhibits much lower leakage current at the voltage of <2.0 V and a slightly higher current density and radiant emittance at a given voltage than that of the device based on untreated CdTe/CdSe QDs. For example, the maximum radiant emittance of 53 mW/cm^2^ was obtained at the voltage of 6.3 V for the device based on untreated CdTe/CdSe QDs. While, for the device based on chloride treated QDs, the same value of radiant emittance could be achieved just at the voltage of 5.1 V, and the maximum radiant emittance increased by ~24.5%, up to 66 mW/cm^2^. Simultaneously, the NIR QLEDs based on untreated CdTe/CdSe QDs exhibit the maximum EQE of 5.7% at the current density of 37.5 mA/cm^2^, as shown in Figure 3B. Moreover, this efficiency increases to 7.1% at 37.5 mA/cm^2^, as well, the maximum EQE is 7.2% for the devices based on chloride treated QDs, which is more than 26.3% higher than that of untreated CdTe/CdSe QDs. Remarkably, EQE of > 5% can be maintained in the range of 0.3–250 mA/cm^2^, which indicates that the efficiency roll-off has been suppressed to some extent, compared to that of device based on transition metal complex. This improved performance can be attributed to the chloride treatment that not only increases the carrier mobility and reduces the carrier accumulation, but also increases the probability of electron-hole radiative efficiency within QD layers.

Figure 3C shows the PL spectra of chloride treated CdTe/CdSe QDs with PL at 788 nm in solution and electroluminescence (EL) spectra of corresponding devices. Compared to the PL spectra, the EL spectra have a red shift of ~8 nm, which is attributed to the combination of interdot interactions within the closely packed QD solids, and the strong electric field that acts to decrease the energy of exciton recombination through the Stark effect (Bae et al., 2013; Mashford et al., 2013). The FWHM of EL spectra increases by ~12 nm, but the parasitical emission cannot be observed, which suggests the excitons have been well-confined in the QD layers. As the voltage increases, the EL peak red shifts ~7 nm owing to the quantum-confined Stark effect (QCSE), but without obviously broadened FWHM.

**Figure 4:**
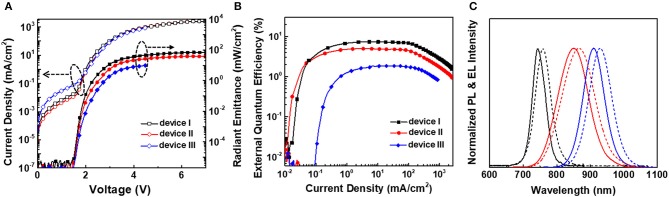
**(A)** Characteristics of current density-voltage-radiant emittance of NIR LEDs based on chloride treated CdTe/CdSe QDs with PL peak of 744, 852, and 910 nm. **(B)** Dependence of external quantum efficiency on the current density of the corresponding devices. **(C)** Normalized PL spectra (solid lines) and EL spectra (dashed lines) of these QLEDs with different emission wavelengths. Herein, device I, II, and III is corresponding to the chloride treated QDs with PL peak of 744 (black), 852 (red), and 910 nm (blue), respectively.

Based on the optimized conditions, chloride treated CdTe/CdSe QDs with PL peaks at 744, 852, and 910 nm have been used to fabricate QLEDs respectively according to the structure shown in Figure 2A, and the relevant electrical characteristics are provided in Figure 4. The maximum radiant emittance of 69.6, 39.1, and 9.8 mW/cm^2^, and the peak EQE of 7.4, 5.0, and 1.8% can be obtained for the devices based on chloride treated CdTe/CdSe QD with PL peak at 744, 852, and 910 nm, respectively. The turn-on voltage is ~1.5 V for all these devices. Figure 4C exhibits the EL spectra of these devices. It can be seen that the EL spectra show a red-shift of 14–19 nm, and are slightly broadened by ~10 nm. The corresponding performance parameters of these devices are summarized in Table 1.

**Table 1:** Summary of performance parameters of NIR QLEDs based on chloride treated CdTe/CdSe with different emission wavelength.

**Device**	**λ_*EL*_ (nm)**	**FWHM (nm)**	***V_***T***_* (V)**	**max. radiant emittance (mW/cm^**2**^)**	**max. EQE (%)**
Device I	758	55	1.5	69.6	7.4
Device II	867	113	1.5	39.1	5.0
Device III	929	80	1.5	9.8	1.8

*Herein, device I, II, and III is corresponding to the chloride treated QDs with PL peak of 744 (black), 852 (red), and 910 nm (blue), respectively*.

## Conclusion

In summary, we have demonstrated highly efficient NIR QLEDs based on chloride treated CdTe/CdSe type-II QDs. After chloride treatment, the maximum radiant emittance and peak EQE increase by 24.5 and 26.3%, up to 66 mW/cm^2^ and 7.2%, respectively, for the devices based CdTe/CdSe with PL peak at 788 nm. Particularly, EQE of > 5% can be maintained at the current density of 0.3–250 mA/cm^2^, which indicates the suppressed efficiency roll-off as compared with that of transition metal complex-based NIR LEDs. This improved performance is mainly ascribed to the chloride surface ligand, which increases the carrier mobility and reduces the carrier accumulation, and increases the probability of electron-hole radiative efficiency within QD layers. Correspondingly, the maximum EQE of 7.4, 5.0, and 1.8% can be achieved for the devices based on chloride treated CdTe/CdSe QDs with PL peaks of 744, 852, and 910 nm, respectively. These results offer the possibility of chloride treated type-II CdTe/CdSe QDs for the applications in the NIR LEDs.

## Data Availability Statement

All datasets generated for this study are included in the article/Supplementary Material.

## Author Contributions

HF and BS synthesized materials and characterized the QDs. JS and QL fabricated devices and collected the performance data of QLEDs. QL and HW wrote the manuscript. HS provided the idea. HW, HS, LL, and ZD helped to modify the manuscript.

## Conflict of Interest

The authors declare that the research was conducted in the absence of any commercial or financial relationships that could be construed as a potential conflict of interest.
