# Supplementary material for: Highly Efficient Near-Infrared Light-Emitting Diodes Based on Chloride Treated CdTe/CdSe Type-II Quantum Dots
Source: Front Chem. 2020 Apr 17;8:266. doi: 10.3389/fchem.2020.00266 (PMC7182655; doi:10.3389/fchem.2020.00266)
Supplement: Supplementary file 1 [file Table_1.DOCX]

Supplementary Material

**Highly Efficient Near-Infrared Light-Emitting Diodes Based on Chloride Treated CdTe/CdSe Type-II Quantum Dots**

Huwei Feng, Jiaojiao Song, Bin Song, Qingli Lin*, Huaibin Shen, Lin Song Li, Hongzhe Wang*, Zuliang Du

Key Lab for Special Functional Materials, Ministry of Education, National and Local Joint Engineering Research Center for High-Efficiency Display and Lighting Technology, School of Materials Science and Engineering, and Collaborative Innovation Center of Nano Functional Materials and Applications, Henan University, Kaifeng 475004, China

***Correspondence:**

Dr. Qingli Lin; Hongzhe Wang

E-mail: qingli1112@126.com

E-mail: [whz@henu.edu.cn](mailto:whz@henu.edu.cn)

**Experimental Section**

***Synthesis of type II CdTe/CdSe with PL peaks at 744, 788, 852 and 910 nm***

Type-II structured CdTe/CdSe core/shell QDs were synthesized by modifying the method previously reported by our group. Detailed preparation is provided as follows.

***Stock solutions for Cd and Te precursors***

Te precursor: Te (0.255 g, 2 mmol) and TOPO (7.7326 g, 20 mmol) were loaded in a 100 mL three-neck flask and degassed for 20 min at 120 °C. Then, 8.9 mL (20 mmol) TOP was added into the above mixture and heated to 320 °C under nitrogen for one hour. During this period, the color of the mixture changed from dark to light yellow, and then, this Te precursor was maintained at 50 °C for further use.

***Synthesis of CdTe QDs***

CdO (0.256 g, 2 mmol), 2 mL oleic acid and 18 mL ODE were added to a three-neck bottle and degassed for 20 minutes at 130 °C. Then, the reaction temperature was raised to 310 °C. 1 mL Te precursor was quickly added and the temperature was lowered to 260 °C for the core growth. Aliquots were taken at different time intervals, UV-vis and photoluminescence (PL) spectra were recorded for each aliquot. The synthesis was carried out under nitrogen flow. Without further purification, this CdTe solution would be directly used to synthesize CdTe/CdSe QDs.

***Stock solutions for Cd, Se precursors for CdSe shell growth***

Stock solution for Cd precursor: A mixture of CdO (0.768 g, 6 mmol), ODE (54 mL) and OA (6 mL) was loaded in a 100 mL three-neck flask and kept at 150 °C under nitrogen flow for 30 min. Subsequently, the solution was heated to 240 °C for 30 min until it turned optically clear and colorless.

Stock solution for Se precursor: Se (0.158 g, 2 mmol) and 20 mL of TOP were mixed into a 50 mL flask and stirred under the nitrogen to prepare the Se precursor solution.

***Synthesis of CdTe/CdSe core/shell QDs with different PL emission peaks.***

The CdTe/CdSe core/shell QDs with different emission wavelengthes were obtained by coating different thickness of CdSe shells onto the CdTe cores at 260 °C with a slightly modified successive ionic layer adsorption and reaction (SILAR) method, through sequential addition of the above Se precursor and Cd precursor solutions.

For CdTe/CdSe core/shell QDs with PL peak at 744 nm, a typical synthesis was performed as follows: 0.2 mL Cd precursor solution was added to the CdTe core solution of 260 °C drop by drop. Five minutes later, 0.2 mL Se precursor was dropwise added to the former core solution. The first coating was completed after 5 min and CdTe/CdSe QDs with PL peak at 744 nm were obtained.

For the synthesis of CdTe/CdSe QDs with PL peaks of 788, 852 and 910 nm, after the first CdSe shell coating, 0.2 mL Cd precursor and 0.2 mL Se precursor were dropwise added into the reaction system successively at intervals of 10 min. The CdTe/CdSe QDs with PL peaks of 788, 852 and 910 nm can be prepared after two, four and six layers of shell growth, respectively.


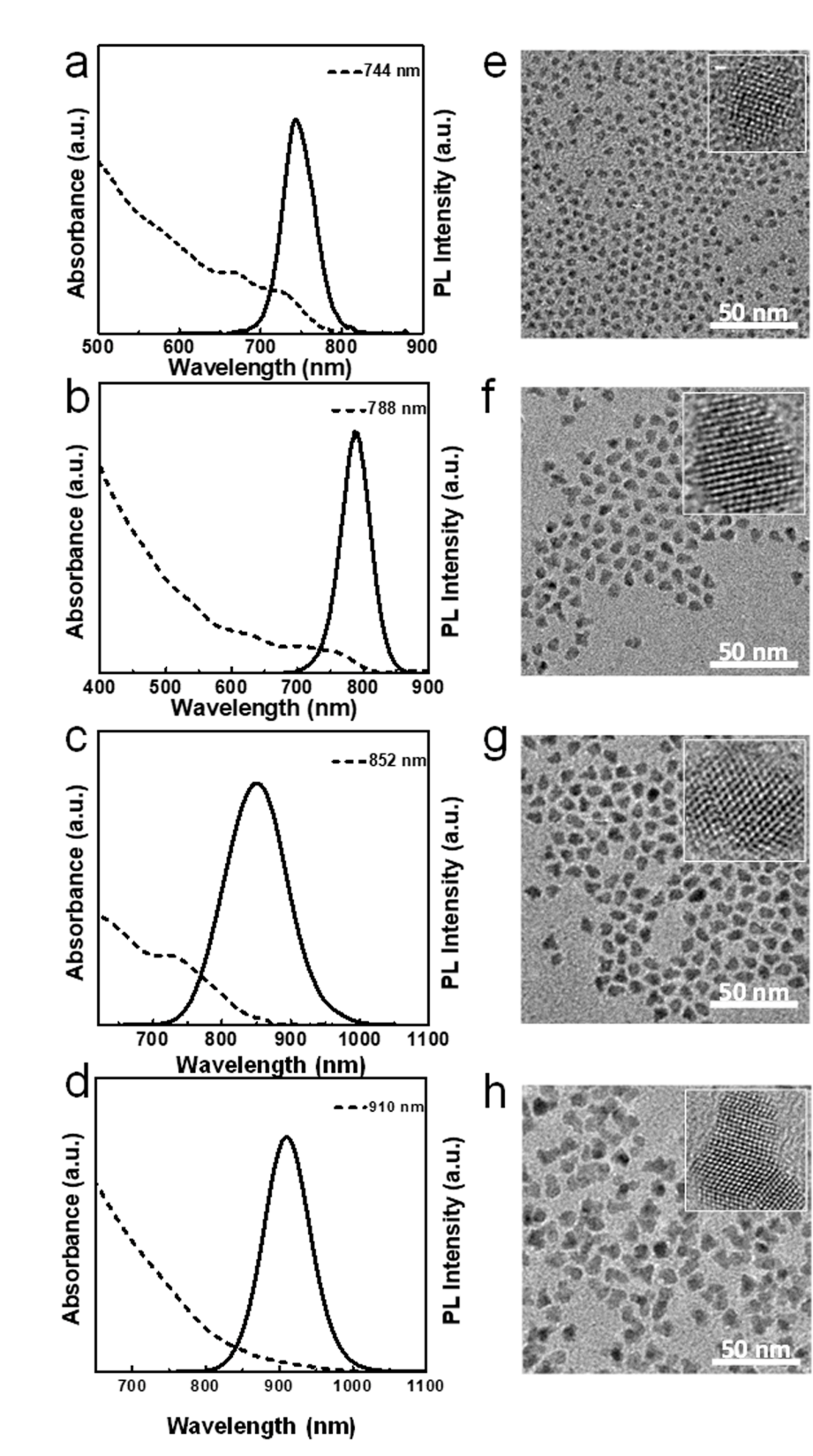


**Supplementary Figure 1.** (a-d) Optical absorption and photoluminescence spectra of type-II CdTe/CdSe QDs, and (e-h) TEM images and high-resolution (HRTEM) images (inset) of type-II structured CdTe/CdSe QDs with PL peaks located at 744, 788, 852 and 910 nm, respectively.


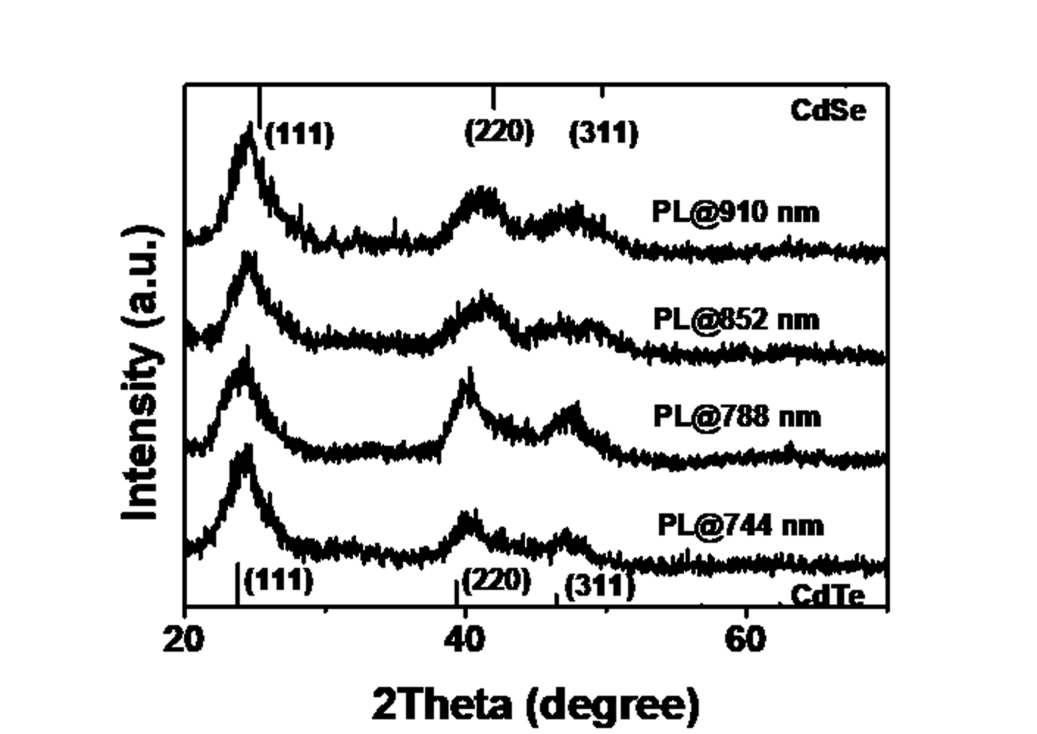


**Supplementary Figure 2.** XRD patterns of as-prepared CdTe/CdSe QDs with different PL peaks.

**Supplementary Table 1.** PL Lifetime of CdTe/CdSe QD solution before and after chloride treatment. The PL decay curves were fitted to a tri-exponential function to investigate the exciton dynamics of the QD solution. The intensity-weighted average exciton lifetime (τ_avr_) was B_1_τ_1_ + B_2_τ_2_ + B_3_τ_3_, where B_1_, B_2_ and B_3_ are fractional intensities and τ_1_, τ_2_ and τ_3_ are lifetimes.

|  | τ_1_ (ns) | τ_2_ (ns) | τ_3_ (ns) | B_1_ (%) | B_2_ (%) | B_3_ (%) | τ (ns) |
| --- | --- | --- | --- | --- | --- | --- | --- |
| Untreated | 42.6 | 5.7 | 84.1 | 58.9 | 1.6 | 39.5 | 58.4 |
| Cl treated | 41.3 | 4.4 | 86.5 | 63.9 | 3.4 | 32.7 | 54.7 |
